# Indole and p-cresol in feces of healthy subjects: Concentration, kinetics, and correlation with microbiome

**DOI:** 10.3389/fmmed.2022.959189

**Published:** 2022-09-21

**Authors:** Francesco Candeliere, Marta Simone, Alan Leonardi, Maddalena Rossi, Alberto Amaretti, Stefano Raimondi

**Affiliations:** ^1^ Department of Life Sciences, University of Modena and Reggio Emilia, Modena, Italy; ^2^ Biogest Siteia, University of Modena and Reggio Emilia, Reggio Emilia, Italy

**Keywords:** indole, p-cresol, uremic toxins, microbiota, metagenome

## Abstract

Indole and p-cresol are precursors of the most important uremic toxins, generated from the fermentation of amino acids tryptophan and tyrosine by the proteolytic community of intestinal bacteria. The present study focused on the relationship between the microbiome composition, the fecal levels of indole and p-cresol, and their kinetics of generation/degradation in fecal cultures. The concentration of indole and p-cresol, the volatilome, the dry weight, and the amount of ammonium and carbohydrates were analyzed in the feces of 10 healthy adults. Indole and p-cresol widely differed among samples, laying in the range of 1.0–19.5 μg/g and 1.2–173.4 μg/g, respectively. Higher fecal levels of indole and p-cresol were associated with lower carbohydrates and higher ammonium levels, that are markers of a more pronounced intestinal proteolytic metabolism. Positive relationship was observed also with the dry/wet weight ratio, indicator of prolonged intestinal retention of feces. p-cresol and indole presented a statistically significant negative correlation with OTUs of uncultured Bacteroidetes and Firmicutes, the former belonging to *Bacteroides* and the latter to the families Butyricicoccaceae (genus *Butyricicoccus*), Monoglobaceae (genus *Monoglobus*), Lachnospiraceae (genera *Faecalibacterium*, *Roseburia*, and *Eubacterium ventriosum* group). The kinetics of formation and/or degradation of indole and p-cresol was investigated in fecal slurries, supplemented with the precursor amino acids tryptophan and tyrosine in strict anaerobiosis. The presence of the precursors bursted indole production but had a lower effect on the rate of p-cresol formation. On the other hand, supplementation with indole reduced the net rate of formation. The taxa that positively correlated with fecal levels of uremic toxins presented a positive correlation also with p-cresol generation rate in biotransformation experiments. Moreover other bacterial groups were positively correlated with generation rate of p-cresol and indole, further expanding the range of taxa associated to production of p-cresol (*Bacteroides*, *Alistipes*, *Eubacterium xylanophylum*, and *Barnesiella*) and indole (e.g., *Bacteroides, Ruminococcus torques, Balutia, Dialister, Butyricicoccus*). The information herein presented contributes to disclose the relationships between microbiota composition and the production of uremic toxins, that could provide the basis for probiotic intervention on the gut microbiota, aimed to prevent the onset, hamper the progression, and alleviate the impact of nephropaties.

## Introduction

Indole and p-cresol are precursors of toxic metabolites, also referred to as uremic toxins, that are eliminated by healthy kidneys *via* the urine and, in case of renal disease, are related to many complications (Vanholder et al., 2018). Among many uremic toxins, indole and p-cresol present the common feature to be products of bacterial metabolism that occurs mostly in the colon by members of gut microbiota.

Indole derives from metabolism of the essential amino acid L-tryptophan by bacterial tryptophanase. Together with its derivatives indole propionic acid, indole acetic acid, skatole, and tryptamine, are potent bioactive metabolites that affect intestinal barrier integrity and immune cells. Intestinal bacteria can directly influence the type and level of tryptophan-derived metabolites, that participate to intracellular signaling within the microbial community (Lee and Lee, 2010) and can activate pregnane X and aryl hycrocarbon receptors, affecting the mucosal immune response and integrity (De Juan and Segura, 2021). Indoxyl sulphate acts as a protein-bound uremic toxin, whose excretion is reduced in patients with Chronic kidney disease (CKD), with severe consequences in terms of systemic toxicity. p-Cresol is the product of the breakdown of tyrosine by intestinal bacteria (Scott et al., 2013; Saito et al., 2018). It.is another protein-bound uremic toxin accumulating in the body of patients with compromised renal function. For both indole and p-cresol, due the cytotoxic effects, CKD and patients in hemodialysis are at increased risk of vascular damage, morbidity, and mortality.

Humans and the gut microbiota evolved together, establishing a close symbiotic interrelationship, fruitful for both. The human colonic microbiota is a dense and complex community of commensal microbes, mostly bacteria, implicated in a number of biological processes such as resistance to colonization (Ruan et al., 2020), immune system modulation (Saldana-Morales et al., 2021), synthesis of essential vitamins and nutrients (Oliphant and Allen-Vercoe, 2019), and breakdown of undigested polysaccharides and proteins (El Kaoutari et al., 2013; Huang et al., 2017; Raimondi et al., 2021). Gut microbes encode a broad diversity of enzymes capable of processing foreign compounds (e.g., dietary compounds, phytochemicals, environmental pollutants, pharmaceuticals, and other xenobiotics), among which those that transform intermediates of protein breakdown into indole, p-cresol, and derivatives, but also those that can participate to degradation of these uremic toxins (Saito et al., 2018; Popkov et al., 2022).

The microbial composition of intestinal microbiota presents a wide diversity in terms of species composition, and, as a consequence, of metabolic potential. Different microbiota is expected to impact on abundance and diversity of enzymes involved in both biosynthesis and degradation of uremic toxins, with major systemic effects associated to different level of these circulating metabolites and, as a matter of fact, alterations in the gut microbiota in patients with CKD can contribute to CKD progression through different mechanisms, including the increased production of microbiota-derived uremic solutes (Vaziri et al., 2013; Wong et al., 2014; Caggiano et al., 2020; Kim and Song, 2020). Nonteheless, these uremic toxins reach steady levels in healthy subjects, where they have been hypothesized to exert also beneficial effects for body functioning (Vanholder et al., 2022).

Microbe-related levels of indole and p-cresol are expected to vary between individuals, due to specificities in gut microbiota composition and dietary patterns (Marcobal et al., 2013). Albeit the era of genomics, post-genomics, and metagenomics encourages *in silico* studies that extract information on microbiota role and activities from the huge amount of available data, still functional observations can be obtained by biotransformations studies with fecal cultures (Tomas-Barberan et al., 2014; Amaretti et al., 2015; Quartieri et al., 2016a).

Little is known about differences of gut microbial populations in the ability to produce and degrade uremic toxins, and a rigorous investigation focused on these biotransformations has not yet been published. The present study seeks to fill this gap. The efficiency of transformation of the precursors tryptophan and tyrosine into indole and p-cresol, and of the degradation of these uremic toxins was compared in fecal cultures inoculated with different intestinal populations provided by healthy subjects. The study was integrated with bacterial composition of fecal inoculum by16 S rRNA gene profiling, and by characterization of fecal samples in terms of dry and wet weight, pH, levels of indole, p-cresol, ammonium, total carbohydrates, and soluble carbohydrates.

## Materials and methods

### Fecal samples and slurries preparation

Fecal samples were obtained from ten healthy adults (five men and five women aged 25–50 years) that provided written informed consent, according to the experimental protocol that was approved with ref. No. 125–15 by the local research ethics committee (Comitato Etico Provinciale, Azienda Policlinico di Modena, Italy). Subjects did not take prebiotics and/or probiotics for the previous 2 weeks or antibiotics for at least 3 months prior to sample collection. Hereinafter, the subjects are referred to as V1–V10. V1 provided 5 fecal samples (referred to as V1a–e), longitudinally collected across a time span of 4 months and spaced at least 7 days from each other. V2 to V10 provided only one sample each.

Fecal samples were collected fresh, sealed within anaerobic plastic bags (AnaeroGen, Oxoid, Basingstoke, United Kingdom), and transferred in a glove box (Concept Plus, Ruskinn Technology, Ltd., Bridgend, United Kingdom) with an atmosphere of 85% N_2_, 10% CO_2_, and 5% H_2_. Within the anerobic cabinet, the feces were homogenized (10%, w/v) in sterile phosphate buffered saline pH 6.5 (PBS, Na_2_HPO_4_ 1 g/L, KCl 0.1 g/L, KH_2_PO_4_ 0.8 g/L, NaCl 8 g/L) or sterile water. PBS suspensions were utilized to for bioconversion experiments. Water suspensions were utilized for the analysis of volatilome, indole and p-cresol, total and soluble carbohydrates, dry weight, pH, and ammonium.

All the chemicals were purchased from Sigma-Aldrich (Merck KGaA, Darmstadt, Germany), unless otherwise stated.

### Chemical analysis

The volatilome was determined by solid-phase microextraction (SPME) followed by GC–MS analysis. 2 mL of water suspension of feces were supplemented with 10 µL of 10 M of HCl within a 10-mL vial, the headspace of which was put in contact with a divinylbenzene/carboxen/polydimethylsiloxane fiber (DVB/CAR/PDMS Supelco; Sigma-Aldrich, St. Louis, MO, United States) for 1 h at 60°C. The VOCs were desorbed and separated in a GC–MS apparatus (7820–5975; Agilent Technologies, Santa Clara, CA, Untied States) equipped with a DB-624 column (30 m ✕ 250 mm ✕ 1.4 mm, Agilent Technologies), according to the conditions reported by Raimondi et al. (2019). The identity of the compounds was inferred from the comparison of their mass spectra with NIST 14 spectral library. The compounds whose identity designation presented a quality score > 95% were listed in the volatilome. The peak areas of compounds were taken to be proportional to their abundances. Ammonium was quantified in samples supernatant using the phenol nitroprusside colorimetric assay, based on the formation of the indophenol blue (Amaretti et al., 2019). Total and soluble carbohydrates were analyzed with anthrone colorimetric assay (Amaretti et al., 2019). Indole and p-cresol were quantified by HPLC (1100 System, Agilent Technologies), equipped with a diode array detector and a C18 Kinetex column (Phenomenex, Torrance, CA, United States) as reported by Amaretti et al. (2019). The dry weight of fecal samples, expressed as the g of dry matter per g of wet feces, was measured with a moisture analyser MBG (VWR International s.r.l., Italy).

### Bioconversion experiments

PBS fecal suspensions were supplemented with the precursor amino acids tryptophan and tyrosine (TT slurries) or the products indole and p-cresol (IP slurries) at the concentration of 200 mM, and were incubated anaerobically at 37 °C for 24 h. Control slurries (C) without any supplement were incubated under the same conditions. Samples were collected at 0, 4, 8, and 24 h of incubation to analyze indole and p-cresol concentration by HPLC.

### 16 S rRNA gene profiling

Total DNA was extracted from 2 mL of water suspension utilizing the kit QiAmp PowerFecal DNA (Qiagen, Hilden, Germany), according to the manufacturer’s protocol. DNA concentration was adjusted to 5 ng/μL after quantification with a Qubit 3.0 fluorimeter (Thermo Fisher Scientific, Waltham, MA, United States). The regions V1-V3 of 16 S rRNA gene sequences were sequenced by Eurofins Genomics (Ebersberg, Germany) using a MiSeq platform (Illumina Inc., San Diego, CA, United States). Raw sequences were analyzed with QIIME 2.0 version 2021.4 (Bolyen et al., 2019), with CUTADAPT and DADA2 plugins for trimming and denoising into amplicon sequence variants (ASVs) (Martin 2011; Callahan 2016). Taxonomy was assigned with VSEARCH (Rognes et al., 2016), utilizing as reference database SILVA SSU release 138 (https://www.arb-silva.de/download/arb-files/) with similarity threshold set at 0.97.

The appropriate QIIME2 plugins were utilized to compute the alpha- (Chao1, Shannon, and Pielou’s evenness) and beta-diversity (Bray-Curtis and Jaccard). Principal Coordinate Analysis (PCoA) was computed with QIIME2, based on the beta-diversity distance matrices.

Correlation between bacterial taxa and VOCs and between taxa and indole/p-cresol transformation values were determined by computing Spearman correlation coefficients (*p* < 0.05) using R package psych. Cytoscape was utilized to visualize correlation networks (Shannon et al., 2003).

PICRUSt2 (Douglas et al., 2020) predicted the abundance of key enzymes in indole e p-cresol biosynthesis, tryptophanase [EC:4.1.99.1] (Yanofsky, 2007) and 2-iminoacetate synthase [ThIH; EC4.1.99.19] and 4-hydroxyphenylacetate decarboxylase [HpdB; EC:4.1.1.83] (Saito et al., 2018; Challand et al., 2010), respectively. Pearson correlation (*p* < 0.05) between the predicted abundances and the and indole/p-cresol transformation values were computed with R package psych.

Aliquots of 5 mL from a fresh culture (16 h) of *B. pseudocatenulatum* WC 0408 were centrifuged and the pellets were washed with PBS buffer pH 6.5. The washed pellets were then resuspended 1:1 in PBS buffer (pH 6.5) supplemented with 1 mM indole. Resting cells were incubated at 37 °C in anaerobic conditions for 48 h and the supernatant was immediately frozen at −20 °C.

### Screening of *Bifidobacteria* and *Lactobacillaceae* for bioconversion of indole and p-cresol

*Bifidobacteria* and *Lactobacillaceae* (33 and 26 strains, respectively; Supplementary Table S1) were grown on MRS (BD Difco, Sparks, United States) for 16 h, at 37°C in anaerobiosis. Biomass was harvested by centrifugation, washed, and resuspended in 50 mM PBS pH 6.5 at a normalized concentration corresponding to 20 OD_600_. Indole and p-cresol were separately added at the final concentration of 1.0 mM and resting cells were incubated for 48 h at 37°C in anaerobiosis. At the end of biotransformation, samples were centrifugated, biomass was extracted with methanol, supernatants and extracts were analyzed with HPLC. Experiments were performed in triplicate.

## Results

### Coprometry, chemical analysis of fecal samples

Fecal samples from 10 healthy adults, *i.e.*, the 5 samples longitudinally collected from the subject V1 and the single ones collected from the other, were analyzed to determine the inter- and intra-individual variability of parameters related to the proteolytic and saccharolytic metabolism, such as pH, content of ammonium, total and soluble carbohydrates, p-cresol and indole, and the dry/wet weight ratio (Table 1, Supplementary Figure S1, S2).

**TABLE 1:** Chemical characterization of fecal samples collected from 10 different volunteers. Feces of Volunteer 1 were collected five times in a period of 4 month (V1a-e). The values are means of 3 replicated measurements ± standard deviation and refer to the wet weight of the feces.

Sample	Dry weight/Wet weight	pH	NH^+^_4_	Total carbohydrates (mg/g_wet_)	Soluble carbohydrates	p-cresol (µg/g_wet_)	Indole (µg/gwet)
**V1a**	0.27 ± 0.02	6.2 ± 0.1	0.42 ± 0.02	6.7 ± 0.7	1.1 ± 0.1	78.6 ± 0.3	17.3 ± 0.2
**V1b**	0.28 ± 0.02	6.5 ± 0.2	0.29 ± 0.03	12.5 ± 1.5	2.7 ± 0.8	35.6 ± 0.9	27.5 ± 0.6
**V1c**	0.31 ± 0.00	7.2±0.1	0.43±0.01	9.7 ± 2.8	1.0 ± 0.2	64.7 ± 1.3	12.4 ± 1.2
**V1d**	0.29 ± 0.01	7.3 ± 0.1	0.46 ± 0.02	22.2 ± 4.0	3.0 ± 1.6	173.4 ± 1.9	12.0 ± 0.3
**V1e**	0.30 ± 0.01	7.0 ± 0.1	0.30 ± 0.04	12.3 ± 1.1	1.7 ± 0.1	73.5 ± 1.0	8.8 ± 0.1
**V2**	0.14 ± 0.03	6.4 ± 0.2	0.07 ± 0.00	17.7 ± 0.2	5.8 ± 0.5	45.5 ± 0.9	6.3 ± 0.8
**V3**	0.18 ± 0.01	6.8 ± 0.2	0.11 ± 0.01	29.5 ± 0.7	2.6 ± 0.2	20.5 ± 0.3	13.6 ± 0.3
**V4**	0.21 ± 0.00	5.7 ± 0.1	0.48 ± 0.03	15.2 ± 0.2	2.6 ± 0.0	41.6 ± 0.3	7.1 ± 0.5
**V5**	0.16 ± 0.02	7.1 ± 0.1	0.15 ± 0.02	36.8 ± 0.9	3.2 ± 0.2	52.0 ± 2.1	19.5 ± 1.2
**V6**	0.10 ± 0.01	6.4 ± 0.1	0.04 ± 0.02	22.9 ± 2.0	7.7 ± 1.9	3.1 ± 0.3	1.0 ± 0.1
**V7**	0.17 ± 0.02	6.4 ± 0.2	0.04 ± 0.03	26.2 ± 2.2	3.2 ± 0.5	1.2 ± 0.1	4.6 ± 0.1
**V8**	0.18 ± 0.02	6.7 ± 0.1	0.16 ± 0.02	18.5 ± 1.2	4.5 ± 0.4	36.7 ± 0.2	11.9 ± 0.3
**V9**	0.23 ± 0.01	6.9 ± 0.1	0.16 ± 0.04	18.2 ± 7.2	2.7 ± 2.8	54.3 ± 0.3	8.8 ± 0.1
**V10**	0.21 ± 0.01	7.0 ± 0.1	0.16 ± 0.02	15.1 ± 0.6	4.8 ± 2.2	50.9 ± 0.3	4.6 ± 0.1
**mean**	0.22 ± 0.07	6.7 ± 0.4	0.23 ± 0.16	18.8 ± 8.1	3.3 ± 1.8	52.3 ± 41.8	11.1 ± 6.9

In the cohort of healthy subjects, the dry/wet ratio of feces (w/w) ranged from 0.10 to 0.31, with a mean value of 0.22. This parameter was significantly higher (*p* < 0.01) in the 5 samples from V1 than in the samples from the other subjects (mean = 0.29 and 0.18, respectively), the latter presenting a greater dispersion (σ_V1a-e_ 0.016 vs. σ_V2-V10_ 0.040). Ammonium content spread over a range of more than one magnitude (0.04–0.48 mg/g). The samples V1a–e were characterized by a significantly higher (*p* < 0.01) ammonium level with respect to the samples V2–V10 (mean = 0.38 vs*.* 0.15 mg/g), but the intra-individual variability within V1 was similar to the inter-individual one between the samples V2–V10. Unlike the dry weight and ammonium, all the other measured parameters did not significantly differ in V1 with respect to the other subjects. Moreover, V1 intra-individual variabilities were similar to the inter-individual ones among V2–V10, with the sole exception of p-cresol (σ_V1a-e_ 52.1 μg/g vs*.* σ_V2-V10_ 21.6 μg/g).

The pH of all the samples was comprised from 5.7 to 7.3, with a mean value of 6.7. Total and soluble carbohydrates ranged from 6.7 to 36.8 mg/g (mean 18.8 mg/g) and from 1.0 to 7.7 mg/g (mean 3.3 mg/g), respectively. The feces contained very different amounts of indole and p-cresol, with both intra- and inter-individual high variability of concentration of these metabolites. Indole ranged from 1.0 to 27.5 μg/g (mean 11.1 μg/g) and values of p-cresol content were spread among more than two orders of magnitude, ranging from 1.2 to 173.4 μg/g (mean 52.3 μg/g).

The level of dry matter in the feces positively and strongly correlated with the content of ammonium (r = 0.80; Table 2) and was negatively linked to the content of total (r = −0.62) and soluble (r = −0.80) carbohydrates. Accordingly, also ammonium negatively correlated with total and soluble carbohydrates (r = −0.56 and −0.67, respectively). Also the fecal content of the uremic toxins was linked to the above mentioned parameters. In particular, p-cresol positively correlated with the dry/wet weight ratio (r = 0.70) and the ammonium content (r = 0.66) and was negatively associated with the content of total and soluble carbohydrates (r = −0.56 and −0.59, respectively). Total and soluble carbohydrates were in countertrend also with the content of indole (r = −0.53).

**TABLE 2:** Pearson correlation (coefficient r) between the chemical parameters measured for the characterization of the fecal samples. *p < 0.05; **p < 0.01.

	Dry weight/Wet weight	pH	NH^+^_4_	Total carbohydrates	Soluble carbohydrates	p-cresol	Indol
**Dry weight/ Wet weight**		0.34	0.80**	−0.62*	−0.80**	0.70**	0.45
**pH**	0.34		−0.01	0.21	−0.15	0.26	0.12
**NH^+^_4_**	0.80**	−0.01		−0.56*	−0.67**	0.66**	0.45
**Total carbohydrates**	−0.62*	0.21	−0.56*		0.32	−0.56*	−0.05
**Soluble carbohydrates**	−0.80**	−0.15	−0.67**	0.32		−0.59*	−0.53*
**p-cresol**	0.70**	0.26	0.66**	−0.56*	−0.59*		0.32
**Indole**	0.45	0.12	0.45	−0.05	−0.53*	0.32	

### Biotransformations

To determine the kinetics of formation and/or degradation of indole and p-cresol by fecal bacteria, bioconversion experiments were carried out with resting cells of intestinal microbiota without any addition (controls C), spiked with indole and p-cresol (samples IP), or supplemented with their precursors tryptophan and tyrosine (samples TT; Table 3).

**TABLE 3:** Accumulation of p-cresol and indole in the fecal slurries. The reported values represent the highest rate observed in the first 8 h of incubation and are expressed as µg of product accumulated per hour per Gram of feces (wet weight). Significantly different mean values compered to the control are indicated; *p < 0.05; **p < 0.01.

Sample	p-cresol (µg/h/g_feaces_)			Indole (µg/h/g_feaces_)		
	C	+IP	+TT	C	+IP	+TT
**V1a**	6.0	4.1	15.5	0.9	−1.4	13.5
**V1b**	6.9	4.6	13.4	1.1	−3.8	10.9
**V1c**	4.4	5.3	8.9	0.5	0.2	12.3
**V1d**	7.7	5.4	31.2	1.6	1.8	15.0
**V1e**	3.2	5.2	12.8	0.8	−1.5	11.4
**V2**	1.9	3.3	13.5	1.0	−3.0	34.5
**V3**	3.2	2.6	28.7	0.8	−0.5	37.4
**V4**	6.8	4.4	14.6	1.0	−1.1	19.3
**V5**	3.9	−0.7	18.5	1.8	−3.6	37.7
**V6**	0.0	0.0	0.3	2.1	−0.3	22.6
**V7**	0.3	1.0	0.1	1.7	1.2	11.1
**V8**	5.1	2.4	21.6	0.4	−1.5	37.5
**V9**	6.0	8.1	23.8	1.7	1.1	30.6
**V10**	6.0	3.2	30.4	0.7	−0.6	36.0
**mean**	4.4	3.5	16.7**	1.1	−0.9**	23.6**

**FIGURE 1:**
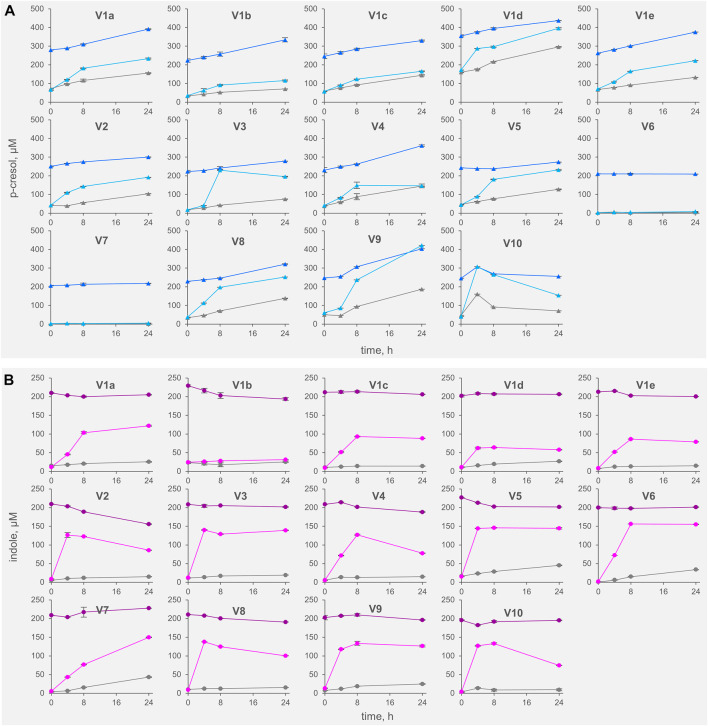
Kinetics of accumulation of p-cresol **(A)** and indole **(B)** in the fecal slurries. The values are means of 3 replicated measurements ± standard deviation. Grey, Controls; dark colors, IP series spiked with indole and p-cresol; light colors, TT series supplemented with tyrosine and tryptophan.

All the controls except V6 accumulated p-cresol with a rate comprised from 0.3 to 6.9 μg/h/g_feces_ (mean 4.4 μg/h/g_feces_). The intra-individual variability within V1was lower than the inter-individual variability observed among V2-V10 (σ_V1a-e_ 1.8 μg/h/g_feces_ vs*.* σ_V2-V10_ 2.5 μg/h/g_feces_). In IP samples, the supplementation of p-cresol did not affect the rate of its accumulation (mean = 3.5 μg/h/g_feces_), compared to the controls (*p* > 0.05). On the other hand, the presence of the precursor tyrosine in TT samples determined a significant increase in the rate of p-cresol production (mean 16.7 μg/h/g_feces_, *p* < 0.01). In samples V6 and V7, accumulation of p-cresol was low or negligible and was scarcely affected by the initial spike of p-cresol (IP) or by the presence of tyrosine (TT). The kinetics of p-cresol concentration during the biotransformations was different among the samples (Figure 1A), with the majority of the samples characterized by the highest rates of accumulation in the first 4–8 h, followed by a constant but slower increase of concentration up to 24 h. Conversely, in a few samples (V3, V4, and V10) a plateau was reached after 4–8 h, then the concentration decreased.

The rate of indole accumulation in C samples was lower compared to that of p-cresol (mean 1.1 μg/h/g_feces_) and was comprised in a narrower range (0.4–2.1 μg/h/g_feces_). V1 intra-individual variability and V2-V10 inter-individual variability were similar. Indole supplementation determined a significant decrease in kinetic of accumulation, with 10 out of 14 IP samples showing a net reduction of indole concentration during incubation. On the other side, the supplement of tryptophan accelerated the indole accumulation up to 37.7 μg/h/g_feces_. In both IP and TT samples, the initial supplement increased the variability of the accumulation rate of indole, that lay in the range of −3.8–1.8 and 10.9–37.7 μg/h/g_feces_, respectively. For indole, the subjects were characterized by different kinetics during the biotransformation, in particular in the TT series (Figure 1B). Most of the TT samples showed the highest rates of accumulation in the first 4–8 h, followed by a slow decrease of the concentration during the last hours of incubation. In some samples (V2, V4, V8, and V10) indole concentration decreased faster, whereas, in two samples (V1a and V7) indole accumulated for 24 h.

### VOCs

**FIGURE 2:**
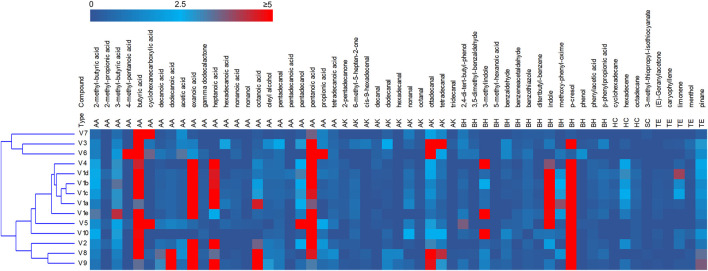
Heatmap of the main VOCs identified by HS-SPME-GC-MS analyses of fecal samples. The relative abundance in the gas chromatogram are reported as shades from blue to red. Only the VOCs occurring at least in two samples are reported. AA acids, alcohols and esters; AK aldehydes ketones; BH benzenoids and heterocycles; HC hydrocarbon; SC solphur containing; TE terpene.

The headspace of the fecal samples analyzed by SPME-GC-MS yielded a total 57 volatile organic compounds (VOCs) occurring in at least 2 samples, 35 of which contributing at list once for > 1% of the relative abundance (Figure 2, Supplementary Figure S3). The main VOCs, occurring most frequently and abundantly, were metabolites derived from the degradation of aromatic amino acids (p-cresol, indole, and 3-methylindole), organic acids (mainly short chain and branched chain fatty acids with 4–8 carbon atoms deriving from the saccharolytic metabolism of sugars and from degradation of aliphatic amino acids), and fatty aldehydes (tetra- and otta-decanal).

PCA of VOCs profiles revealed that the intra-individual variability was less pronounced than the interindividual one (Supplementary Figure S4). The samples V1a–e closely distributed in the lower and right quadrant with V4 and V5, all characterized by high values of aromatic amino acids metabolites. V2, V8, and V9 lay separately and were characterized by short chain fatty acids, whereas the cluster including samples V3, V6, and V7 was featured by high amounts of cyclohexane carboxylic acid, butyric acid and hexadecanal.

### 16 S rRNA gene profiling

A metataxonomic survey of microbiota used as inoculum was carried out by 16 S rRNA gene profiling. Only the first and the last samples of V1 longitudinal series (V1a and V1e) were analyzed and sample V5 was excluded due to errors in the sequencing procedure. A total of 566,443 sequence reads was obtained, 28,119–182,257 per sample. The reads were dereplicated into 2,064 ASVs hitting a reference sequence in SILVA database and collapsed at the 7th level of taxonomic annotation into 286 features.

**FIGURE 3:**
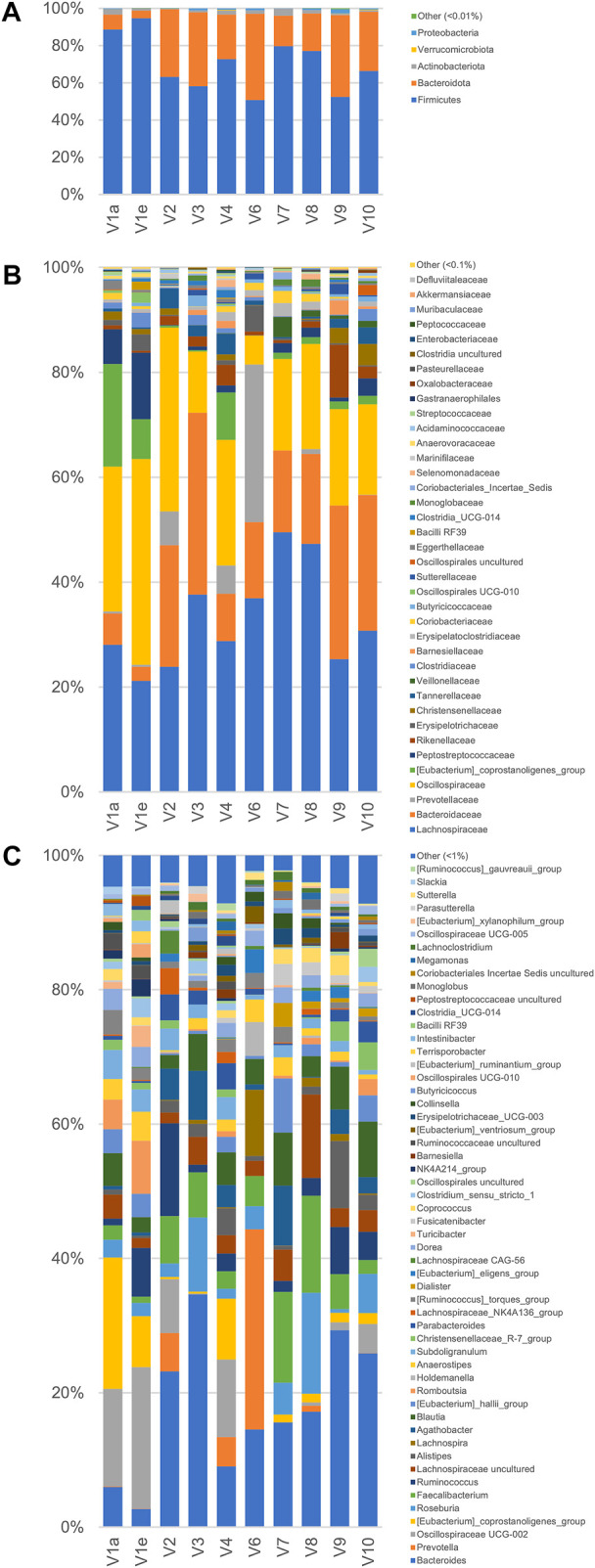
Microbiota composition of the fecal samples analyzed by 16 S rRNA gene profiling. From volunteer 1, only the first and last sample (V1a and V1e), collected 4 months apart, were analyzed. V5 sample sequencing with Illumina platform did not provide useful sequences for analysis. The compositions are reported as relative abundance of the reads collapsed at the level of Phylum **(A)** Family **(B)**, and Genus **(C)**.

As a whole, the fecal microbiota was largely composed by Firmicutes (50.6%–94.6%lower amount of Bacteroidota (4.1%–46.5%, and only minor number of Actinobacteriota (0.2%–3.8%) and Proteobacteria (0.1%–2.3%; Figure 3). Among the bacterial groups recognized as proteolytic (Amaretti et al., 2019; Raimondi et al., 2021), the main taxa were Lachnospiraceae (mean 32.9%), Bacteroidaceae (17.8%), and Ruminococcaceae (13.7%) followed by Peptostreptococcaceae, Clostridiaceae, Streptococcaceae, and Enterobacteriaceae, each of them accounting on average for less than 2.9% of the whole bacterial population.

According to the rarefaction plots of the main alpha diversity indices (Supplementary Figure S5A), the diversity of all samples was entirely captured and sequences were rarefied at the level of the sample with the lowest number of reads (20,251). Non-phylogenetic beta-diversity analysis was performed and reported in Supplementary Figure S5B. Calculating distances among samples with non-quantitative algorithms, the inter-individual distances among the microbiota was higher than the intra-individual one, with V1 samples laying very closely in the PCo1 vs PCo2 plots. On the other hand, with quantitative metrics that calculate distances also considering the abundance of the identified taxa and not only their presence, the samples V1a and V1e lay far from each other, showing that fecal samples from the same subject could be more distant taking into account quantitative composition than samples from different subjects.

PICRUSt2 was utilized to reconstruct the metabolic functions of the microbial communities, with the aim to infer the abundance of the genes encoding the key enzymes involved in metabolism of uremic toxins. The genes encoding tryptophanase, 2-iminoacetate synthase, and 4-hydroxyphenylacetate decarboxylase were pinpointed in all the microbiomes (Supplementary Figure S6). 2-Iminoacetate synthase, involved in transformation of tyrosine to p-cresol, presented similar abundance in all the samples, laying in the range between 0.07% and 0.09% of the relative abundance of all predicted genes, whereas abundance of 4-hydroxyphenylacetate decarboxylase, involved in the formation of p-cresol, and tryptophanase, responsible of the transformation of tryptophan to indole, exhibited wide differences among samples. 4-Hydroxyphenylacetate decarboxylase ranged from 0.0001% to 0.012%, with the lowest and the highest abundance occurring in V2 and V6, respectively. Such marked difference among samples of genes associated to production of p-cresol was non significantly correlated with differences in p-cresol formation rate, in both control and TT slurries. Tryptophanase ranged from 0.001% to 0.014%, with the lowest values occurring in samples V1a and V6 and the highest ones in V3 and V9. Positive significant correlation between the levels of tryptophanase and indole formation rate in TT slurries (r = 0.69) was identified.

### Correlations of microbiome composition with VOCs profiles and with kinetics of p-cresol and indole accumulation

**FIGURE 4:**
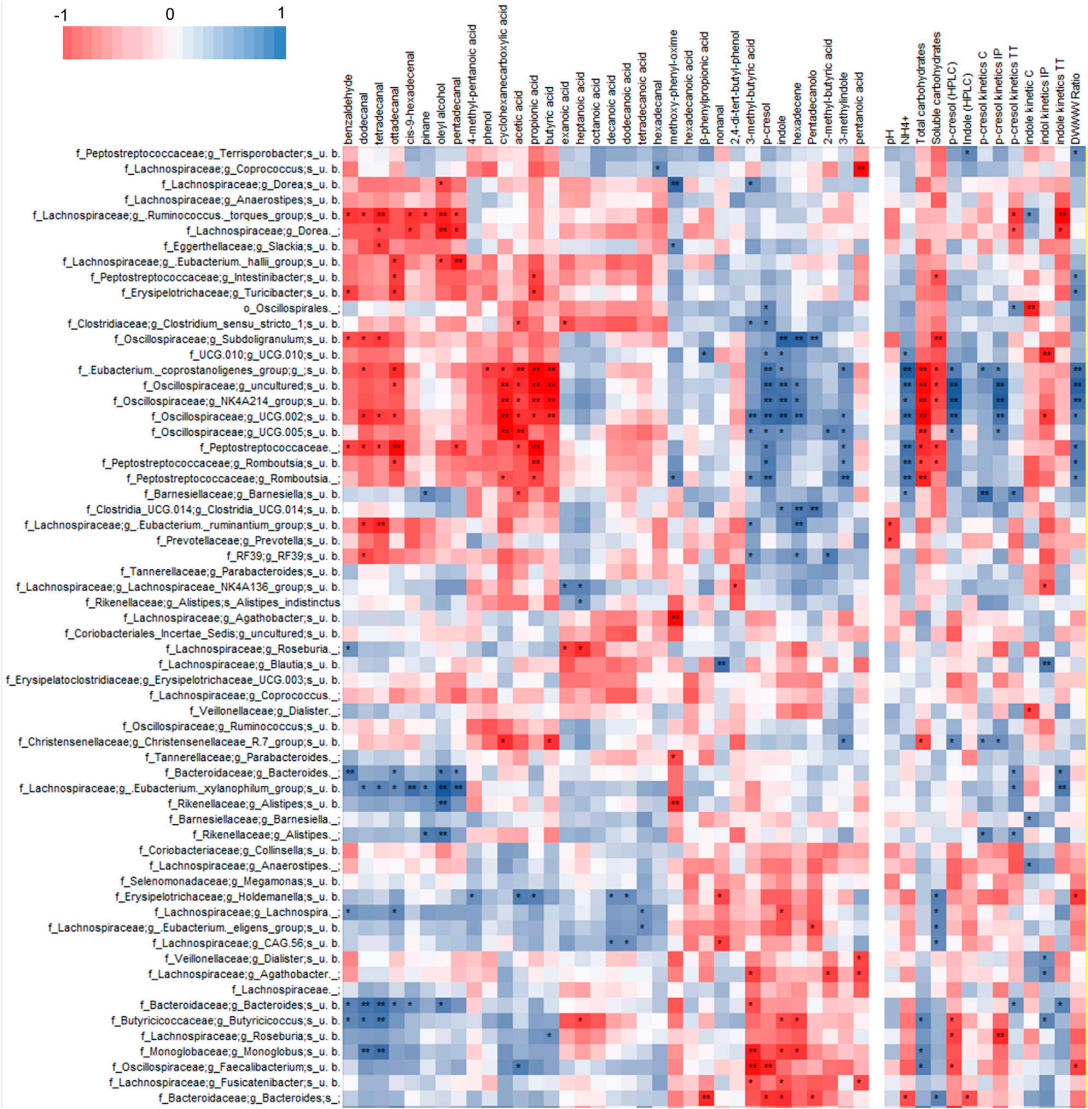
Heatmap of Spearman’s correlation between microbial species, VOCs identified by HS-SPME-GC-MS analyses and coprometry parameters. Microbial taxa and VOCs are filtered to show only features with 1% abundance in at least one subject.

Correlation analysis of VOCs and 16 S rRNA gene profile revealed a statistically significant correlation of p-cresol and indole with some OTUs of Firmicutes (Figure 4, Supplementary Figure S7), mainly uncultured bacteria belonging to the families of Ruminococcaceae (including the genus *Subdoligranulum*), Oscillospiraceae, *Eubacterium coprostanoligenes* group, and Peptostreptococcaceae (genus *Romboutsia*). These bacterial groups also presented a significant positive correlation with 3-methyl indole and other products of proteolytic metabolism, while they were negatively correlated with organic acids (acetic, propionic, and butyric acids), fatty aldehydes, phenol, and benzaldehyde.

P-cresol and indole in both feces and their volatilome presented a statistically significant positive correlation with OTUs of uncultured Bacteroidetes and Firmicutes. The former belonged to the genus *Bacteroides* and the latter to the families Butyricicoccaceae (genus *Butyricicoccus*), Monoglobaceae (genus *Monoglobus*), Lachnospiraceae (genera *Faecalibacterium*, *Roseburia*, and *Eubacterium ventriosum* group), all presenting a general positive correlation (although not always reaching statistical significance) with short chain fatty acids, fatty aldehydes, phenol, and benzaldehyde.

Any correlation between the microbiome composition and the chemical signature of stool samples was searched utilizing Spearman’s test. The analysis revealed that the OTU that positively correlated with uremic toxins generally presented a significant positive correlation with the fecal amount of ammonium and with the dry/wet weight ratio, and a significant negative correlation with both soluble and/or total carbohydrates, whereas they did not present any relationship with the pH.

Oscillospiraceae, *Eubacterium coprostanoligenes* group, and Peptostreptococcaceae that positively correlated with the uremic toxins also presented a general positive correlation with p-cresol generation rate in biotransformation experiments, although the statistical significance was not always reached. Moreover, some other OTUs that did not present any significant correlation with the uremic toxins were positively associated with production rates of p-cresol (e.g., *Bacteroides*, *Alistipes*, *Eubacterium xylanophylum*, and *Barnesiella*) and indole (e.g., *Bacteroides, Ruminococcus torques, Balutia, Dialister, Butyricicoccus*) by the fecal slurries.

### Bioconversion of p-cresol and indole with resting cell of bifidobacteria and lactobacillaceae

**FIGURE 5:**
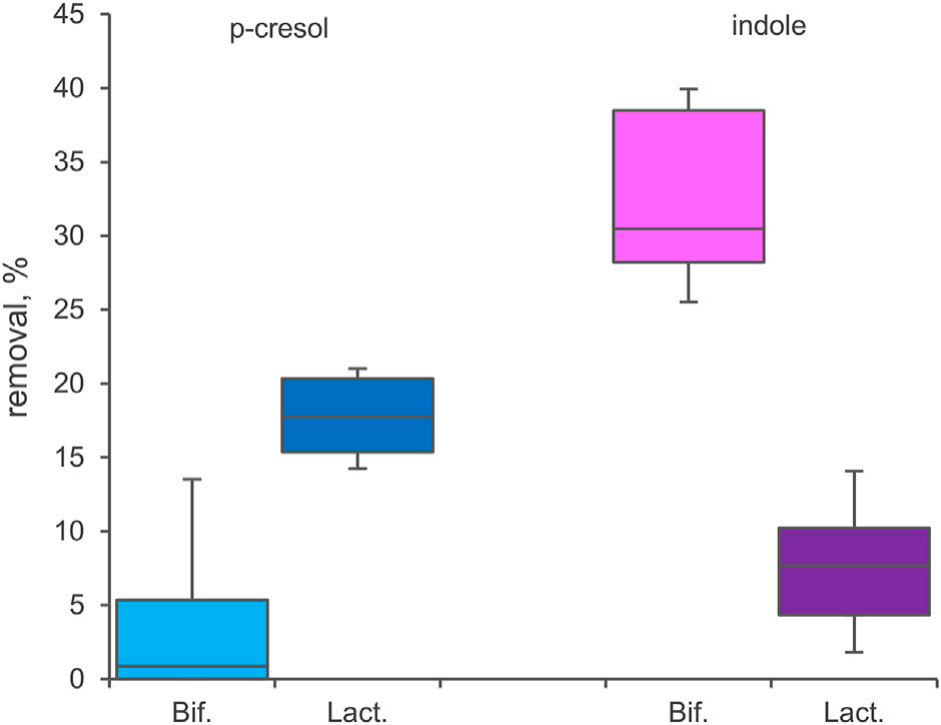
Biotranformation of p-cresol and indole with resting cells of 33 Bifidocateria and 26 Lactobacillaceae strains. Box plot reports the median values, 10th, 25th, 75th, and 90th percentile of the relative amount of p-cresol and indole removed from the supernantat referred to the initial values. Significant differences are indicated. **p < 0.01.

33 *Bifidobacterium* strains belonging to 8 different species or subspecies and 26 Lactobacillaceae ascribed to 15 taxa (Supplementary Table S1) were screened for bioconversion of p-cresol and indole in resting cells condition. The percentage of removal of the two molecules from the supernatants and the absorption in the inoculated biomasses were determined after 48 h of incubation. All the tested bifidobacteria strains were able to reduce indole concentration in the supernatant (Figure 5), with values of removal ranging from 24.0% to 41.7% (mean 32.4%). Among the screened species, *B. longum* (subspecies *longum* and *infantis* comprised) was the most effective in indole removal (mean 36.0%, *p* < 0.05). In the tested condition, bifidobacteria were less prone to reduce p-cresol concentration, with a mean value of 4.2% and higher variability among strains (from 0% to 18.2%). In Lactobacillaceae biotranformations, p-cresol was removed from the supernatant more efficiently than indole (mean values of 17.6% and 7.5%, respectively), the latter also characterized by the higher variability among strains (from 0.8% to 16.3%). The tested species showed no significant differences in the amount of removed p-cresol and indole. Analyzing the biomasses of bifidobacteria and Lactobacillaceae after incubation, always less than 3.0% of absorbed indole or p-cresol was detected.

## Discussion

In this study, the volatilome, the microbiota composition, and the physical and chemical features of feces from healthy subjects were investigated to obtain information on the role of intestinal bacteria in the formation of the uremic toxins indole and p-cresol, providing a preliminary view on the intra- and interindividual variability.

Higher fecal levels of indole and p-cresol were associated with lower carbohydrates and higher ammonium levels, that are markers of a more pronounced intestinal proteolytic metabolism. This is in agreement with the positive relationship observed also with the dry/wet weight ratio, since an increased water absorption results from a prolonged retention of feces within the colon that also leads to the onset of protein breakdown as the availability of fermentable carbohydrates becomes lower (Tottey et al., 2017).

The present study also enabled preliminary considerations on intra- and interindividual variability of markers of proteolytic and saccharolytic metabolism affecting the level of uremic toxins. The 5 samples longitudinally provided by the subject V1 over a period of 4 months indicated that the range of concentration of p-cresol and indole can be extremely wide within the same individual, likely as a result of different component of the diets affecting the availability of precursors, and of the impact of the diet on microbiota composition, in its turn affecting the abundance of producing/degrading bacteria. The features of the fecal samples less influenced over the time were dry weight, according to a personal transit time minimally changing in a healthy status, and total carbohydrates, likely reflecting regular eating habits. Albeit subject V1 likely had his own food habits, variations of pH and ammonium concentration likely corresponded to a different intake of proteins and fibers, with resultant differences in terms of proteolytic and saccharolytic metabolic activity in the colon. Fecal specimens from the other 9 volunteers presented a higher content of water and a lower concentration of ammonium, likely reflecting for V1 a longer transit time, which implicates higher water absorption and a shift towards a proteolytic metabolism of colonic bacteria, following depletion of carbohydrates. Accordingly, the mean levels of indole and p-cresol tended to be lower in V2–V10 subjects, when compared to V1, albeit differences were not statistically significant, likely because of the low numerosity of the samples per group and the large variability within the groups.

Previous studies with enrichment cultures of gut microbiota identified main proteolytic bacteria within Lachnospiraceae, Oscillospiraceae, Clostridiaceae, Eubacteriaceae, Peptostrocaccaceae, Sutterellaceae, and Enterobacteriaceae and revealed that several OTUs of these families correlated with the accumulation of indole and p-cresol within cultures (Amaretti et al., 2019; Raimondi et al., 2021). Some families encompassing potential proteolytic taxa were remarkably abundant in the present study, such as Lachnospiraceae and Ruminococcaceae that represented the 32.9% and 21.7% on average, respectively. On the other hand, other proteolytic families were minor components of the microbiota, with Eubacteriaceae (*Eubacterium corpostanoligenes* group), Peptostreptococcaceae, Clostridiaceae, and Enterobacteriaceae being on average the 4.2%, 2.9%, 1.1%, and 0.1%, respectively.

The headspace of fecal samples contained a mixture of linear and branched SCFA, aromatic compounds, aldehydes, and terpenes. Major markers were organic acids, including butyric and propanoic acids, whereas acetic acid was the less abundant. A high level of indole was a feature of some fecal samples, whereas, with the exceptions of specimens V6 and V7, abundant p-cresol was detected in the headspace of all the specimens. Comparison of VOCs and 16 S rRNA gene profiles pointed out that specific OTUs ascribed to Lachnospiraceae, Oscillospiraceae, and Peptostreptococcaceae presented positive correlation with fecal levels of indole and p-cresol, providing additional evidence of the relationship linking uremic toxins with intestinal protein breakdown and amino acids fermentation. Interestingly, any significant relationship with Enterobacteriaceae, that were major indole producers in fecal cultures, was not found, due to the generally low levels of this family in the samples.

Bioconversions with resting cells obtained from fresh fecal inocula were carried out in order to evaluate the capability of the colonic community to produce or degrade indole and p-cresol and to establish a relationship with bacterial taxa. The presence of the precursors tyrosine and tryptophan significantly increased the accumulation of both the uremic toxins, confirming that colonic bacteria have a strong potential to produce these metabolites. However, the burst of indole production was more marked with respect to p-cresol, suggesting that tryptophan was more limiting in feces compared to tyrosine or that the enzymatic arsenal of gut bacteria was more prone to metabolize this substrate. Interestingly, this attitude was not shared by all the subjects, since V6 and V7 transformations did not efficiently accumulate p-cresol, even in presence of tyrosine. This behavior was consistent with low p-cresol levels in the feces of these subjects, that likely harbored a microbiota with limited metabolic potentials in terms of p-cresol production. The presence of indole reduced its production, suggesting some feedback mechanism depressing indole accumulation when this compound was present in relevant amounts.

Gryp et al. (2020) obtained bacterial isolates belonging to 92 species, spread across several families (mainly within Firmicutes, Bacteroidetes, and Proteobacteria), capable of producing p-cresol under anaerobic conditions from the feces of CKD patients with different severity, indicating that the ability to release uremic toxins is widespread in the microbiome. Coherently, the OTUs that positively correlated with fecal levels of uremic toxins in the present study were mostly identified as uncultured bacteria belonging to Oscillospiraceae, *Eubacterium coprostanoligenes* group, and Peptostreptococcaceae and presented a positive correlation with also p-cresol generation rate in biotransformation experiments. Likewise, other bacterial groups were positively correlated with generation rate of p-cresol and indole, further expanding the range of taxa associated to production of p-cresol (*Bacteroides*, *Alistipes*, *Eubacterium xylanophylum*, and *Barnesiella*) and indole (e.g., *Bacteroides, Ruminococcus torques, Balutia, Dialister, Butyricicoccus*). Although a causal relationship cannot be established from this data, it seems likely that these bacterial groups are either directly involved in p-cresol or indole generation or are involved in protein breakdown, resulting uremic toxins producers from tyrosine and tryptophan.

Bifidobacteria and Lactobacillaceae have been extensively utilized as probiotics to exert beneficial health effect on the host, despite they do not represent a dominant component of the human gut microbiome (Quartieri et al., 2016b; Rossi et al., 2016; Bottacini et al., 2017; Khalesi et al., 2019). Any potential ability to reduce the levels of indole and p-cresol would be of great importance, as would open new applications of specifically designed probiotics in the amelioration of uremic toxin levels. The screening herein presented revealed that the ability to reduce p-cresol and indole from the environment is widespread among Lactobacillaceae and bifidobacteria, even though with wide strain to strain variability. The mechanism of toxins removal of these bacteria remains to be clarified, particularly for the most promising strains belonging to *Bifodobacterium longum* supsb. *longum*, *Lacticaseibacillus rhamnosus*, and *Lactiplantibacillus plantarum*. Interestingly, lactobacilli and bifidobacteria presented complementary effectiveness in the removal of p-cresol and indole, respectively, suggesting a combined utilization in a probiotic formula.

The present study is the first focusing on the levels of p-cresol and indole, the content of other compound derived by microbial metabolism proteins and carbohydrates, the rate of formation/degradation of these uremic toxins and the correlation with microbiota taxa in the feces of healthy subjects. Despite being a small study, with sample size as a main limitation, the information herein presented contributes to disclose the relationships between microbiota composition and the production of uremic toxins. Therefore, it could represent a hypothesis-generating study and inspire larger confirmatory studies devising nutritional interventions, also combining diet modification with probiotics administration, aimed to prevent the onset, hamper the progression, and alleviate the impact of CKD.

## Data Availability

The datasets presented in this study can be found in online repositories. The names of the repository/repositories and accession number(s) can be found below: https://www.ncbi.nlm.nih.gov/, PRJNA843561.
