# Supplementary material for: Indole and p-cresol in feces of healthy subjects: Concentration, kinetics, and correlation with microbiome
Source: Front Mol Med. 2022 Sep 21;2:959189. doi: 10.3389/fmmed.2022.959189 (PMC11285674; doi:10.3389/fmmed.2022.959189)

Indole and p-cresol in feces of healthy subjects: concentration, kinetics, and correlation with microbiome

Francesco Candeliere[1], Marta Simone[1], Alan Leonardi[1], Maddalena Rossi[1,2], Alberto Amaretti[1,2,*], Stefano Raimondi[1,*]

[1]Department of Life Sciences, University of Modena and Reggio Emilia, Modena, Italy

[2]Biogest Siteia, University of Modena and Reggio Emilia, Reggio Emilia, Italy

*** Correspondence:**

Stefano Raimondi
stefano.raimondi@unimore.it

Alberto Amaretti
alberto.amaretti@unimore.it

Supplementary Material

Supplementary Table 1. Biotransformation of p-cresol and indole with resting cells of 33 *Bifidobacterium* strains belonging to 8 different species or subspecies and 26 Lactobacillaceae ascribed to 15 taxa. The percentage of removal of the two molecules from the supernatants were reported as means ± standard deviation of three independent experiments.

strain	p-cresol	indole
Bifidobacterium animalis subsp. lactis WC 0432	5.3 ± 1.3	35.2 ± 8.9
Bifidobacterium bifidum MB 254	0.7 ± 0.2	39.9 ± 5.4
Bifidobacterium bifidum WC 0417	0.9 ± 0.3	29.6 ± 4.0
Bifidobacterium bifidum WC 0418	0.0 ± 0.0	38.5 ± 11.8
Bifidobacterium breve B 2429	0.0 ± 0.0	25.2 ± 7.9
Bifidobacterium breve WC 0420	0.0 ± 0.0	28.2 ± 0.6
Bifidobacterium breve WC 0421	3.0 ± 0.7	26.4 ± 1.8
Bifidobacterium breve WC 0422	0.0 ± 0.0	31.5 ± 0.5
Bifidobacterium breve WC 0423	13.0 ± 1.9	24.0 ± 4.3
Bifidobacterium catenulatum WC 0458	0.7 ± 0.2	36.4 ± 2.7
Bifidobacterium catenulatum WC 0469	0.0 ± 0.0	39.9 ± 5.6
Bifidobacterium lactis WC 0413	0.9 ± 0.3	29.0 ± 5.0
Bifidobacterium lactis WC 0414	0.0 ± 0.0	28.7 ± 0.4
Bifidobacterium lactis WC 0455	1.9 ± 0.2	28.9 ± 12.0
Bifidobacterium lactis WC 0459	13.6 ± 2.3	37.8 ± 3.2
Bifidobacterium longum MB 201	1.3 ± 0.3	26.9 ± 0.1
Bifidobacterium longum MB 219	0.3 ± 0.2	40.0 ± 2.8
Bifidobacterium longum subsp. infantis MB 208	0.0 ± 0.0	41.7 ± 6.0
Bifidobacterium longum subsp. infantis MB 256	3.5 ± 0.7	39.0 ± 3.3
Bifidobacterium longum subsp. infantis WC 0434	0.0 ± 0.0	30.5 ± 4.6
Bifidobacterium longum subsp. longum WC 0436	13.6 ± 2.4	38.0 ± 5.0
Bifidobacterium longum subsp. longum WC 0438	0.0 ± 0.0	39.0 ± 4.2
Bifidobacterium longum subsp. longum WC 0439	13.2 ± 1.5	40.2 ± 3.3
Bifidobacterium longum subsp. longum WC 0440	13.1 ± 0.4	25.3 ± 3.1
Bifidobacterium longum subsp. longum WC 0443	0.0 ± 0.0	39.7 ± 4.8
Bifidobacterium pseudocatenulatum MB 114	18.2 ± 4.5	29.0 ± 3.0
Bifidobacterium pseudocatenulatum MB 116	5.4 ± 1.2	24.5 ± 1.4
Bifidobacterium pseudocatenulatum MB 243	0.0 ± 0.0	31.2 ± 3.4
Bifidobacterium pseudocatenulatum WC 0400	13.8 ± 2.1	28.8 ± 1.4
Bifidobacterium pseudocatenulatum WC 0401	0.0 ± 0.0	30.0 ± 0.3
Bifidobacterium pseudocatenulatum WC 0403	0.0 ± 0.0	26.2 ± 0.9
Bifidobacterium pseudocatenulatum WC 0407	12.9 ± 1.5	26.8 ± 0.7
Bifidobacterium pseudocatenulatum WC 0408	2.5 ± 0.5	31.7 ± 5.2
Lacticaseibacillus paracasei WC 0227	13.1 ± 1.8	4.3 ± 0.7
Lacticaseibacillus rhamnosus WC 0232	15.7 ± 2.0	2.4 ± 1.1
Lacticaseibacillus rhamnosus WC 0295	20.8 ± 1.9	11.2 ± 2.4
Lacticaseibacillus rhamnosus WC0216	11.0 ± 1.6	0.8 ± 1.1
Lactiplantibacillus pentosus WC 0308	15.4 ± 2.2	4.9 ± 2.3
Lactiplantibacillus plantarum WC 0230	19.9 ± 2.0	4.5 ± 2.1
Lactiplantibacillus plantarum WC 0214	16.1 ± 2.1	7.1 ± 3.3
Lactiplantibacillus plantarum WC 0292	21.4 ± 1.9	14.9 ± 2.1
Lactobacillus acidophilus WC 0203	14.4 ± 1.9	9.1 ± 2.3
Lactobacillus acidophilus WC 0281	20.7 ± 1.8	1.3 ± 0.1
Lactobacillus delbrueckii subsp. delbruekii WC 0286	14.0 ± 2.0	0.9 ± 1.2
Lactobacillus delbrueckii subsp. lactis WC 0290	14.9 ± 2.1	16.3 ± 2.9
Lactobacillus delbrueckii subsp. lactis WC 0291	19.6 ± 1.7	9.3 ± 1.7
Lactobacillus gasseri WC 0213	16.9 ± 2.4	13.9 ± 2.6
Latilactobacillus curvatus WC 0301	16.3 ± 1.2	10.9 ± 2.3
Latilactobacillus sakei WC 0313	15.8 ± 2.2	10.5 ± 1.9
Latilactobacillus sakei WC 0314	19.8 ± 1.7	6.8 ± 1.3
Levilactobacillus brevis WC 0282	20.3 ± 1.9	8.5 ± 1.4
Ligilactobacillus ruminis WC 0225	21.3 ± 2.0	6.9 ± 0.7
Limosilactobacillus fermentum WC 0212	15.4 ± 1.2	8.4 ± 2.1
Limosilactobacillus mucosae WC 0229	18.6 ± 2.0	3.8 ± 2.2
Limosilactobacillus reuteri WC 0215	14.7 ± 1.1	5.1 ± 1.7
Limosilactobacillus reuteri WC 0224	20.7 ± 1.9	2.3 ± 0.3
Limosilactobacillus reuteri WC 0231	19.0 ± 2.2	8.2 ± 2.3
Limosilactobacillus reuteri WC 0293	21.4 ± 2.0	14.3 ± 2.5
Limosilactobacillus reuteri WC 0294	20.3 ± 1.9	8.8 ± 1.3

Supplementary Figure 1. Box-plot representation of the chemical parameters measured for the characterization of the fecal samples collected from 10 different volunteers (V1-10). Feces of volunteer 1 were collected five times (V1a-e). The mean values, 10th, 25th, 75th, 90th percentile and the significant differences are reported. * p<0.01

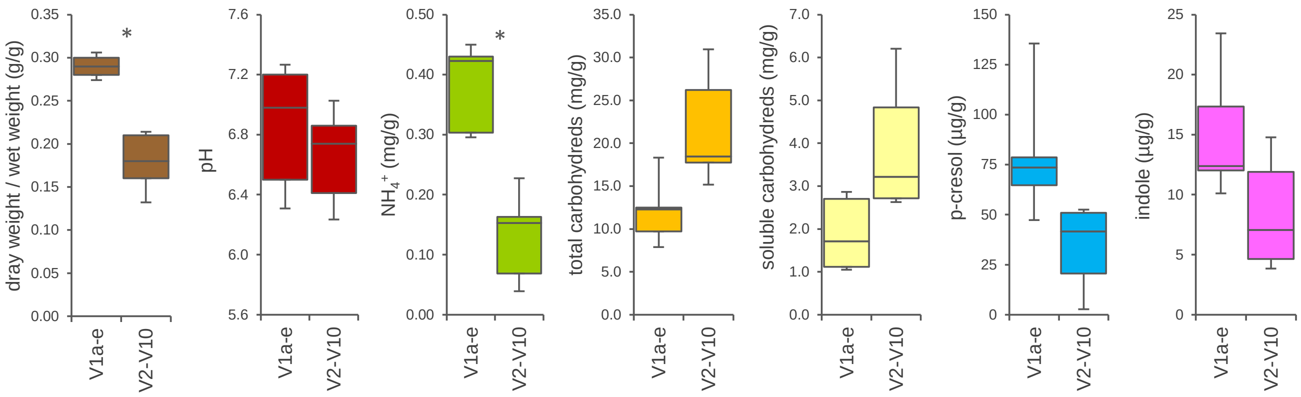

Supplementary Figure 2. Analysis of indole and p-cresol standard (1mM in H_2O) with HPLC, equipped with a diode array detector and a C18 column.

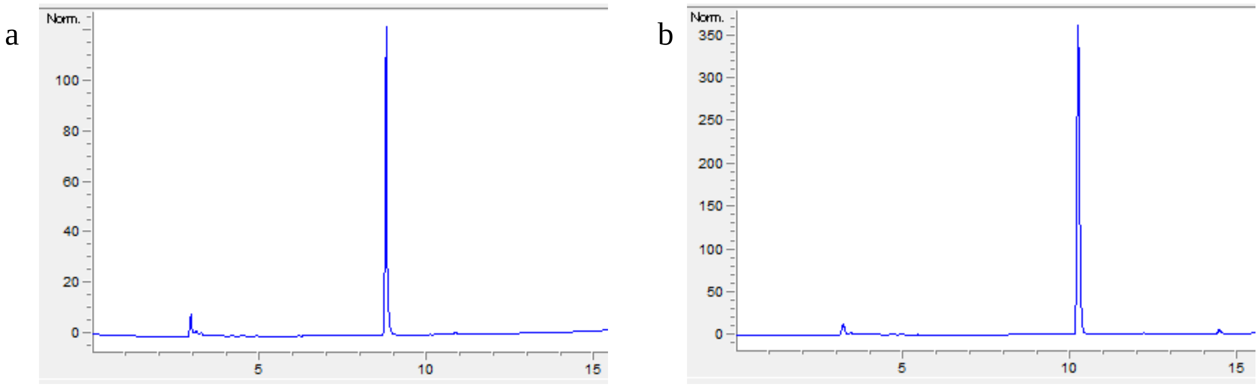

Supplementary Figure 3. Representative chromatogram of the SPME-GC-MS analysis of fecal samples. The main VOCs, occurring most frequently and abundantly are indicated.

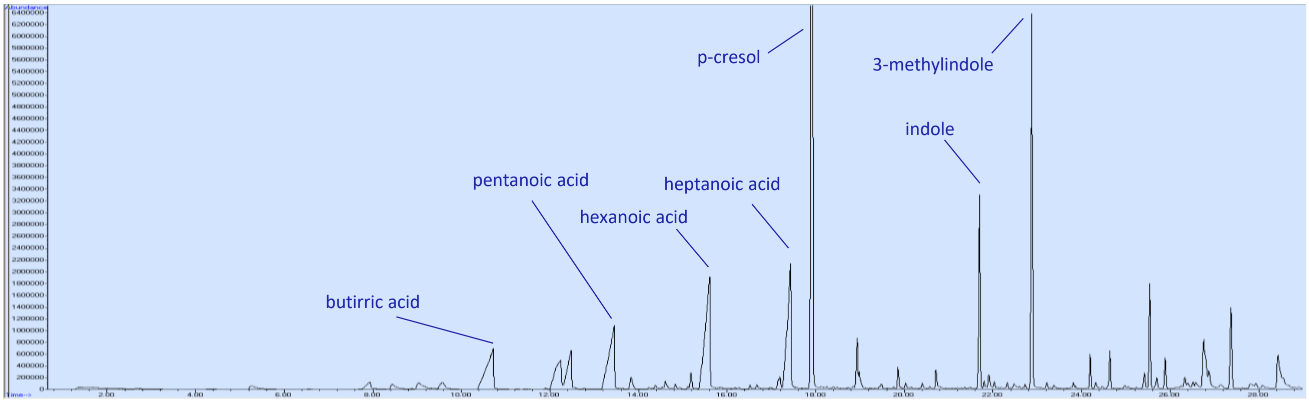

Supplementary Figure 4. PCA analysis of VOCs detected by SPME-GC-MS analyses in the fecal samples. **a** PC1*vs* PC2 plot. Red diamond, samples V1a-e; blue circle, samples V2-V10. Ellipses represent the 95% of variability of the observed parameters ascribed to the corresponding set of samples. **b** Score plot showing the VOCs mainly contributing to PC1 and PC2.

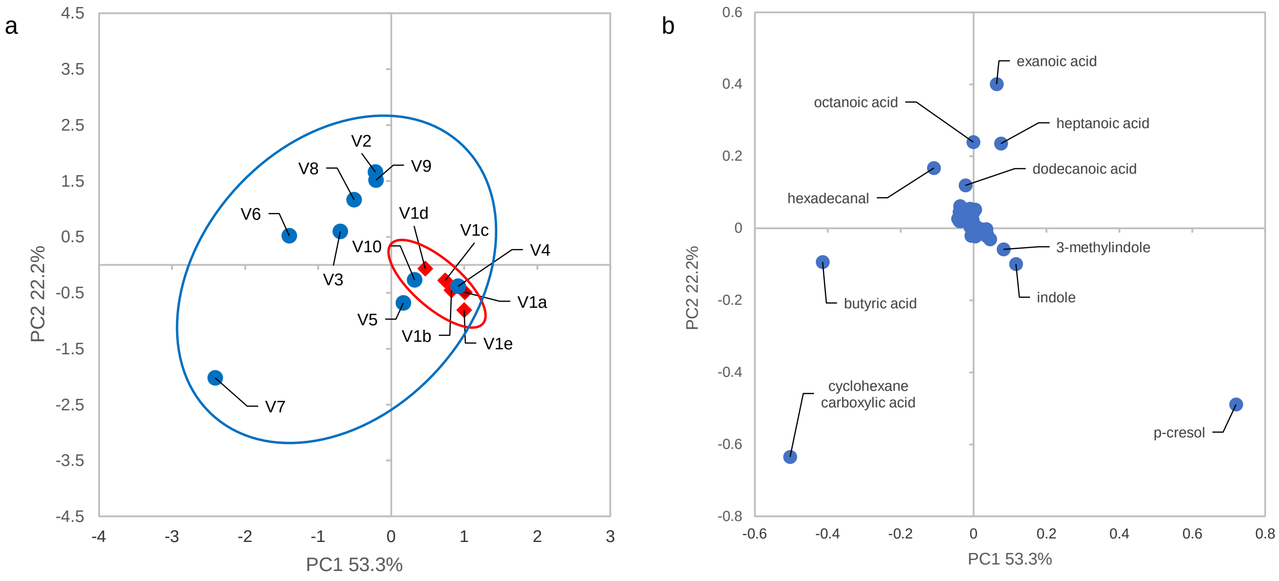

Supplementary Figure 5. Analysis of microbiota composition of the fecal samples obtained by 16S rRNA gene profiling. **a** Alpha rarefaction plots of the main alpha diversity indexes. **b** Beta-diversity analyses: PCoA based on Bray-Curtis (quantitative) and Jaccard (qualitative) distance matrices.

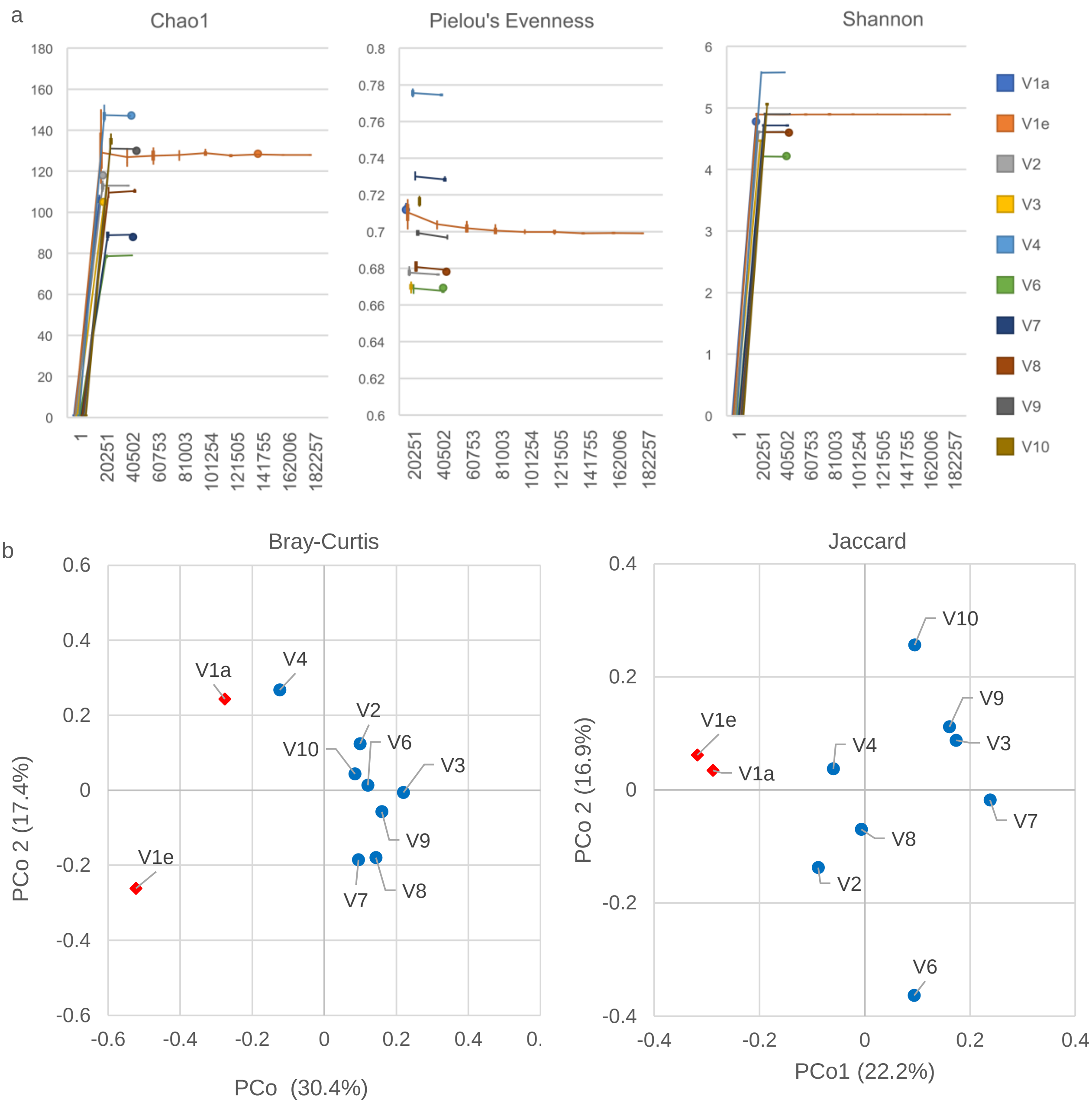

Supplementary Figure 6: **a** Metabolic pathway of tryptophan degradation into indole. **b** Relative abundance of tryptophanase (tnaA) in the metagenomes predicted by PICRUSt2. **c** Pearson's correlation between indole accumulation rate in TT slurries and tryptophanase relative abundance.

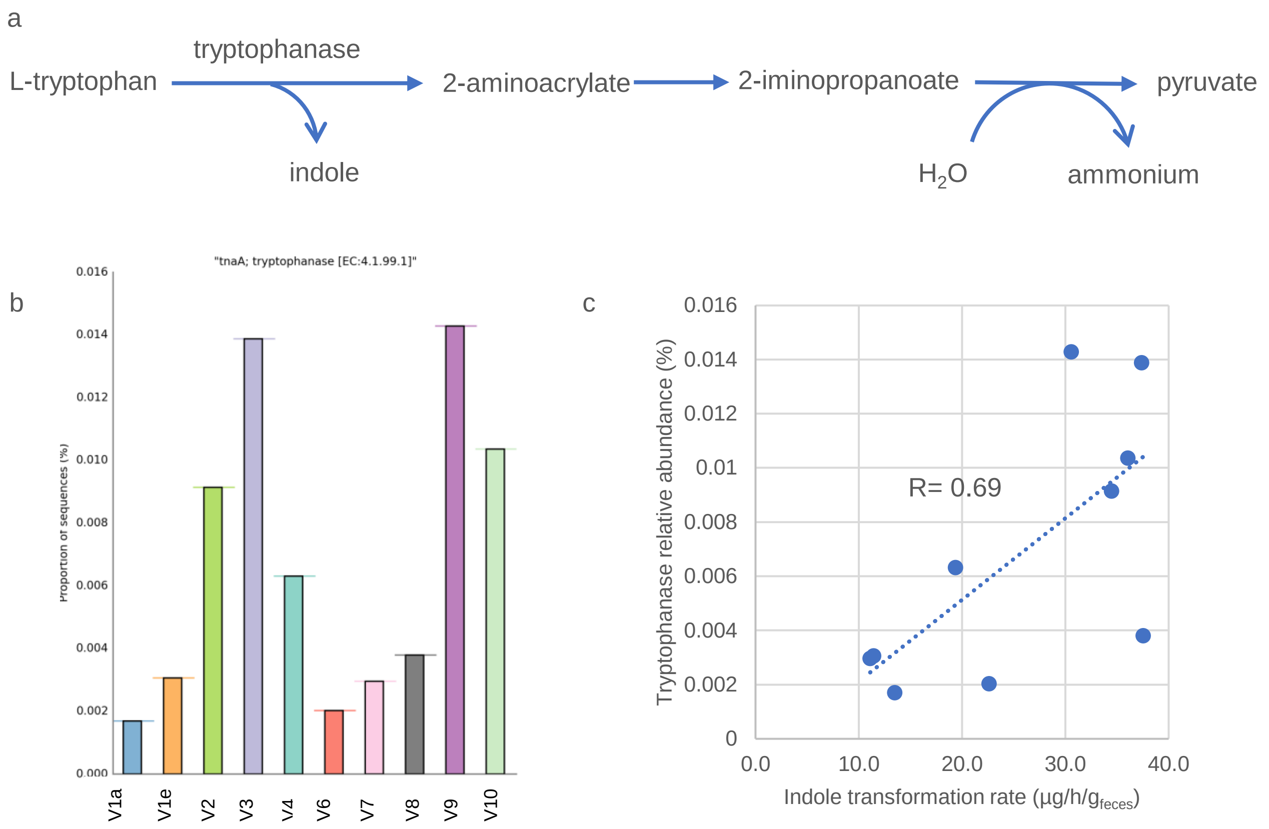

Supplementary Figure 7: Heatmap of Spearman's correlation between microbial species, VOCs identified by HS-SPME-GC-MS analyses and coprometry parameters. Blue cladogram on the right represent features clusters obtained with UPGMA algorithm using PAST software according to their correlation with VOCs.

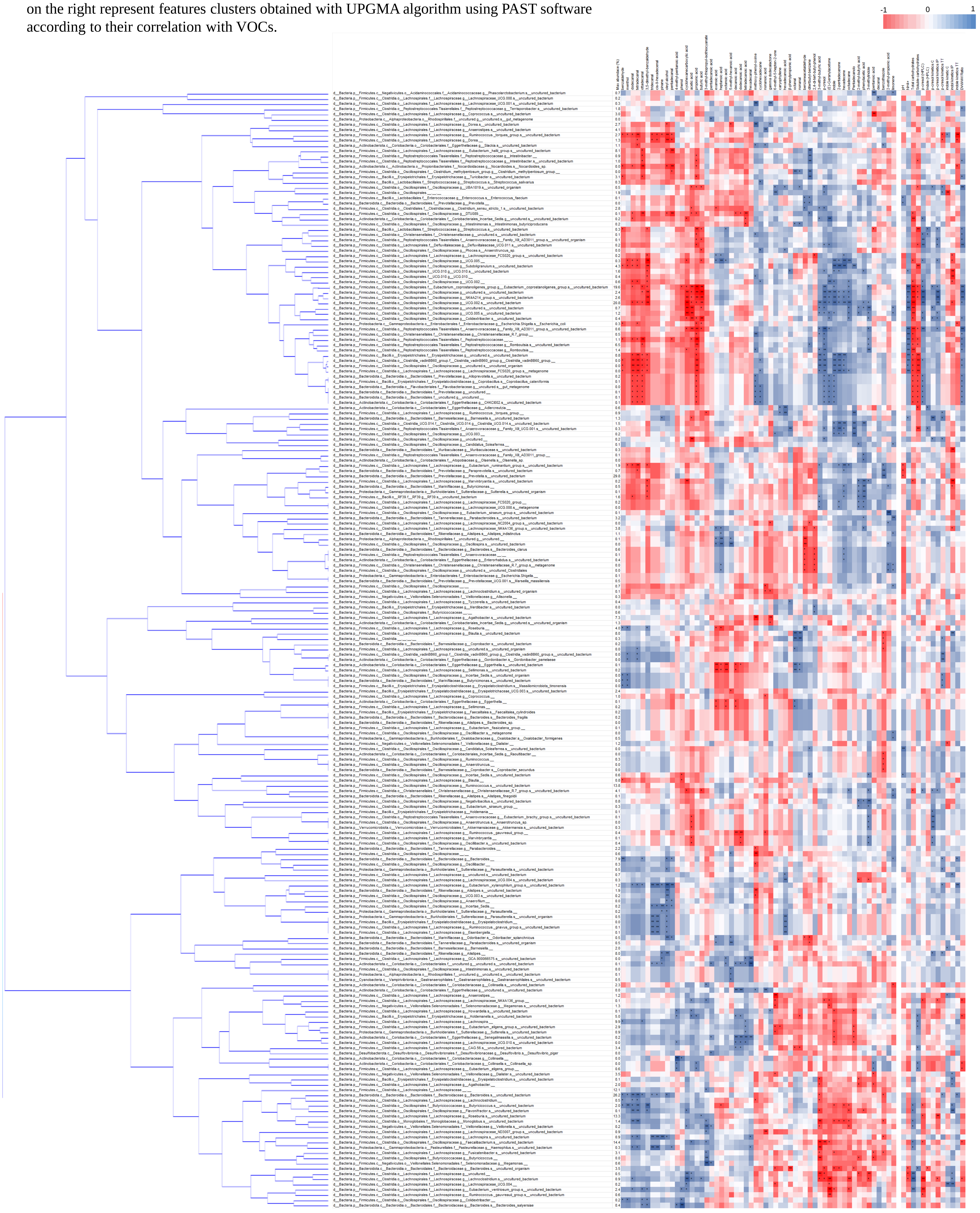

Supplement: Supplementary file 1 [file DataSheet1.PDF]
